# An empirical demonstration of the effect of study design on density estimations

**DOI:** 10.1038/s41598-021-92361-2

**Published:** 2021-06-23

**Authors:** Muhammad Ali Nawaz, Barkat Ullah Khan, Amer Mahmood, Muhammad Younas, Jaffar ud Din, Chris Sutherland

**Affiliations:** 1 Department of Biological and Environmental Sciences, Qatar University, Doha, Qatar; 2 Department of Zoology, Quaid-i-Azam University, Islamabad, Pakistan; 3 Snow Leopard Foundation, Islamabad, Pakistan; 4 Snow Leopard Trust, Seattle, USA; 5 Department of Environmental Conservation, University of Massachusetts, Amherst, MA 01002 USA; 6 Centre for Research Into Ecological & Environmental Modelling, University of St Andrews, St Andrews, Scotland, UK

**Keywords:** Conservation biology, Conservation biology

## Abstract

The simultaneous development of technology (e.g. camera traps) and statistical methods, particularly spatially capture–recapture (SCR), has improved monitoring of large mammals in recent years. SCR estimates are known to be sensitive to sampling design, yet existing recommendations about trap spacing and coverage are often not achieved, particularly for sampling wide-ranging and rare species in landscapes that allow for limited accessibility. Consequently, most camera trap studies on large wide-ranging carnivores relies on convenience or judgmental sampling, and often yields compromised results. This study attempts to highlight the importance of carefully considered sampling design for large carnivores that, because of low densities and elusive behavior, are challenging to monitor. As a motivating example, we use two years of snow leopard camera trapping data from the same areas in the high mountains of Pakistan but with vastly different camera configurations, to demonstrate that estimates of density and space use are indeed sensitive to the trapping array. A compact design, one in which cameras were placed much closer together than generally recommended and therefore have lower spatial coverage, resulted in fewer individuals observed, but more recaptures, and estimates of density and space use were inconsistent with expectations for the region. In contrast, a diffuse design, one with larger spacing and spatial coverage and more consistent with general recommendations, detected more individuals, had fewer recaptures, but generated estimates of density and space use that were in line with expectations. Researchers often opt for compact camera configurations while monitoring wide-ranging and rare species, in an attempt to maximize the encounter probabilities. We empirically demonstrate the potential for biases when sampling a small area approximately the size of a single home range—this arises from exposing fewer individuals than deemed sufficient for estimation. The smaller trapping array may also underestimate density by significantly inflating $\sigma$. On the other hand, larger trapping array with fewer detectors and poor design induces uncertainties in the estimates. We conclude that existing design recommendations have limited utility on practical grounds for devising feasible sampling designs for large ranging species, and more research on SCR designs is required that allows for integrating biological and habitat traits of large carnivores in sampling framework. We also suggest that caution should be exercised when there is a reliance on convenience sampling.

## Introduction

Increased human impacts on the environment have had profound effects on landscapes and the species that occupy them, and large carnivores in particular have undergone dramatic declines globally, leaving some on the verge of extinction^1^. Conserving these iconic and ecologically critical species is the need of the time, requiring effective conservation planning, which itself necessitates robust population monitoring and assessments. However, large carnivores are elusive, exist at low densities, and maintain large home ranges^2,3^ making them notoriously difficult to study.

Camera trapping has revolutionized wildlife monitoring, and in particular the monitoring of large carnivores such as the snow leopard *Panthera uncia*^4,5^ a species that is especially subject to this vast array of monitoring challenges^2^. Snow leopards are a globally imperiled species, and despite a recent and contentious down listing from endangered to vulnerable^6^, there is little doubt that the species is data deficient throughout large parts of its range, meaning that any population status assessment is incomplete at best^6,7^. As a result, global initiatives have been put in place with a specific focus on developing standardized monitoring tools, in an attempt to develop reliable range-wide populations estimates of snow leopards^8^. Camera trapping plays a central role in achieving this conservation goal which is ambitious, but absolutely necessary if the current population declines are to be halted or reversed.

Like many large carnivores, snow leopard monitoring had relied on a range of indirect monitoring methods such as questionnaire surveys, interviews and sign surveys^4,9–11^ which have now been almost completely replaced by more efficient, reliable, and affordable, camera traps^3,12^. However, recent studies indicate a lack of agreement between estimates of densities, even for similar methodological approaches^4,13^. This suggests that, although the choice of specific methodology is obviously a fundamental consideration, the resulting statistical inference is likely sensitive to a range of other factors including, perhaps most importantly, study design^14,15^.

The spatial capture–recapture (SCR) is a relatively new framework for data collection and analysis that is now widely applied and that provides inferences about several aspects of a species’ spatial ecology, including spatially explicit estimates of density^16^. Owing to enormous potential in monitoring large carnivores, SCR is becoming method of choice in ecology. However, the approach is constrained by sample size requirements and study design challenges, as large carnivores often yield sparse data owing to their biological traits (low numbers, large ranges, secretive nature). For example, capture rates in snow leopards are as low as 3/1000 trap-nights^4^. Conversely, conventional CMR literature suggests minimum capture of 10–20 uniquely identified individuals^17^, and desirable detection probability is 0.2^18,19^. It is logistically difficult to achieve such level of detections in low-density wide-ranging populations that exist in rugged and remote habitats.

The study design is often ignored in large carnivores’ studies that implement SCR, due to limited familiarity in field biologists about this relatively new method and deficiency of guidelines for adopting this framework particularly for rugged terrains and elusive species. This is particularly surprising given explicit link between the number and configuration of traps and the quality of the data, and hence the reliability of the inferences made^20,21^. Thus, while sparse and imperfect data is an inevitable outcome in studies of large carnivores, it is critical that the gap in understanding feasible SCR study design for challenging landscapes is addressed. This need stems from the fact that sampling design is a critical factor in population studies^22,23^, and poor designs can lead to serious flaws in the data and consequently impedes utility of such information in management. For example, more than half of the camera trap studies carried out for snow leopards in past 10 years, could not yield abundance estimates owing to poor design^24^.

Recent reviews^14,24,25^ indicate that the majority of camera trap studies on large carnivores adopt convenience or judgmental sampling (e.g.^26,27^). Ideas of optimal sampling design are well developed in context of several field methods. For example, Mackenzie and Royle^28^ provide practical guidance on the efficient design of occupancy in terms of selection of sampling units, timing of repeat surveys and allocation of survey effort. Similarly, theory and sampling frameworks are established in distance sampling which allow practicing ecologists implement such methods effectively in challenging field conditions^29^. Although rules-of-thumb for SCR study design exists about trap spacing and spatial coverage^30–32^, their generality has yet to be tested, and moreover, they represent situations that are unlikely to be attainable in real landscapes, especially for rare and large ranging species (e.g., uniform grids, contiguous habitats, and ignoring terrain limitations, see Dupont et al. (2020).

In this paper we attempt to empirically emphasize the importance of design in SCR monitoring studies, and in particular demonstrate how density estimates of low-density large-ranging species are sensitive to study design. Taking advantage of a fortuitous juxtaposition of extremely different camera trapping arrays (a sparse array over a large area vs. a dense array in a smaller area) deployed in consecutive years in the same landscape, we empirically evaluate the sensitivities of SCR inference. Our hope is to motivate further discussion and development around the issue of SCR study design, especially for more challenging taxa.

## Methods

### Study area

**Figure 1:**
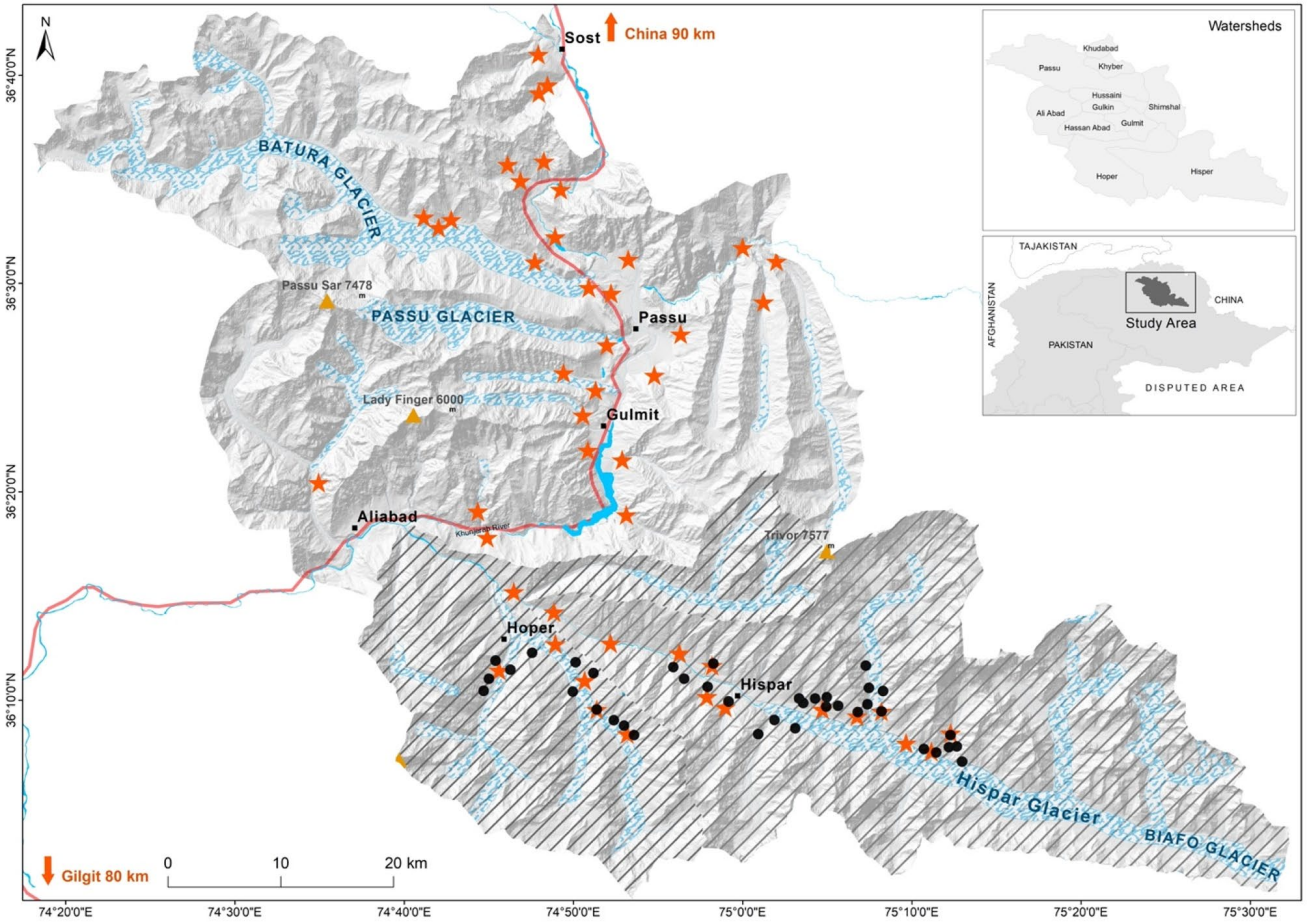
Map the study area showing camera locations of compact (circles) and diffused (stars) designs. Area surveyed during the session 1 (compact design) is hatched. Map is developed in ArcGIS version 10.8 (www.esri.com).

This study was conducted in districts of Hunza and Nagar in Gilgit-Baltistan, Pakistan, encompassing 4100 km^2^ area, across 11 watersheds. It lies in the Karakoram mountain range, and connects two major national parks (Central Karakorum NP, and Khunjerab NP, Fig. 1). Topography is dominated by high-altitude snow-covered mountains, high peaks with steep slopes and extremely rugged terrain. Elevation ranges from 2000 to 7800 m. Climate in the study area varies with altitude: winters are dry and cold with temperatures ranging from − 23 to 10.8 °C; summers are mild with temperatures reaching 34 °C in June–August (Pakistan Meteorological Department, https://www.pmd.gov.pk/en/). Precipitation is mostly (90%) in the form of snow and ranges from 200 to 900 mm annually^33^. Permission was obtained for conducting the study in the aforementioned National Parks.

### Camera trapping

We use data collected from two snow leopard camera trapping studies that overlap spatially but differ markedly in the way cameras were deployed. The two camera trapping sessions did not overlap temporally. The first camera trapping session was conducted from March to May in 2016 and comprised of 38 single-camera trapping stations (Fig. 1). Cameras were deployed so as to maintain approximately 1 km spacing between cameras resulting in a convex hull (a proxy for the spatial extent) of 253 km^2^ and a corresponding trap density of 15 cameras per 100 km^2^. We refer to this session as the *compact* design. The second camera trapping session was conducted from April to June in 2018 and comprised of 44 single-camera trapping stations. In this case, cameras were deployed to maintain approximately 5 km spacing resulting in a spatial extent of 2030 km^2^, almost an order of magnitude larger, and a corresponding trap density of 2 cameras per 100 km^2^. We refer to this session as the *diffuse* design. Given the large-scale ranging behavior of snow leopards, the differences in trap spacing and spatial extent represent adherence (diffuse) or not (compact) to existing recommendations to sample multiple home ranges and to deploy cameras to achieve on average 2 cameras per home range^32^. Thus, for snow leopards with a typical home range of around 200 km^2^^34^, the compact arrangement samples fewer home ranges (i.e., individuals) more intensively, whereas the diffuse configurations sample more individuals, but each is exposed to fewer traps.

In both years, access to large parts of the area was limited by extreme weather, harsh terrain, and excessive snowfall, which resulted in cameras being restricted to areas in proximity to walkable tracks. Areas above 5000 m and areas very close to settlements were not sampled. We used Reconyx (Hyperfire PC 900 and Reconyx Hyperfire HC 500) motion triggered cameras set to take three photos with an interval of 1 s between shots when triggered. Cameras were fixed on metal rod roughly 50 cm above the ground and were orientated to maximize the likelihood of photographing the forehead, limbs, and flanks. Castor and fish oils were used as a lure to increase detection probability^35^. When selecting the specific site, areas with high probability of snow leopard passage were preferred (e.g., narrow ridgelines, bottoms, important connection and junction points between two areas). In order to avoid identification errors, we adopted a two-step individual identification process. First, two teams independently checked all snow leopard photos and assigned individual IDs based on their unique pelage patterns (Supplementary Appendix S2). In the second confirmation step, individual capture histories were compared, and any inconsistencies were resolved though discussion. ArcGIS version 10.8 (www.esri.com) was used to prepare study area map and plot locations of the camera traps (Fig. 1).

### Data analysis

We analyzed the resulting spatially explicit encounter histories using spatial capture-recapture (SCR) methods^16,36^. Because we did not have time-varying covariates, i.e., encounter rate was assumed constant across all camera days, we collapsed the encounter histories to a single trap-specific count with an associated effort variable, the number of days each camera was operational^21^. As a result, we analyzed the data using a standard SCR model in where the encounter rate is modelled using a half normal encounter model:

$$\lambda _{{ij}} ~ = ~\lambda _{0} \times {\text{exp}}\left( { - \frac{{d\left( {s_{i} ,x_{j} } \right)}}{{2\sigma ^{2} }}} \right).$$

The parameter λ_ijk_ is the expected count of individual *i* at camera trap *j*, $d\left( {s_{i} ,x_{j} } \right)$ is the distance between an individual’s activity center ($s_{i}$) and a trap ($x_{j}$), and the parameters $\lambda _{0}$ and $\sigma$ are, respectively, the baseline encounter rate (i.e., expected number of encounters when $d\left( {s_{i} ,x_{j} } \right) = 0$), and the spatial scale parameter that determines the distance over which encounter probability declines. We use the log of the number of operating days as an offset in the baseline parameter of the Poisson encounter rate model:

$${\text{log}}\left( {\lambda _{0} } \right) = \alpha ~ + ~{\text{log}}\left( {operating~days} \right),$$

i.e., effort is included as a covariate with a coefficient fixed at 1 which gives the convenient interpretation of $\lambda$ as a *per day* encounter rate.

Individual activity centers ($s_{i}$) are latent variables that are estimated using a homogeneous Poisson point process model informed by spatial locations of capture. SCR requires an explicit definition of the area over which density is to be estimated, typically represented as a fine mesh or regularly spaced points (the state space) large enough to contain activity centers of all individuals with at least one trap in its home range. Because the two studies were spatially overlapping, and to facilitate the comparison between model outputs, we used the same state space for both years (sessions) which was a represented by a grid of points covering an area defined by a 20 km buffer around all traps locations from both years with a 2.5 km^2^ resolution. Unsuitable snow leopard habitat, large human settlements and areas above 5800 m, were removed from the state space.

Using the multi-session likelihood in the R^37^ package oSCR^38^, we jointly analyzed both years of data, treating each year as a session, which allows for the comparison of models with session-specific parameters with models that assume constant parameters. This resulted in eight possible models: a ‘constant model’ in which density, detection ($\lambda _{0}$) and space use ($\sigma$) are assumed to be constant across years; a ‘session’ model in which density, detection and space use are allowed to vary by year; and all combinations in between (Table 2). We compared the eight models using AIC^39^.

Our focus on empirically evaluating the effect of camera trap array configuration (compact in 2016 vs. diffuse in 2018) on resulting inference is predicated on the assumption that snow leopard densities are the same in both years. We argue that this is a valid assumption for several reasons. First, the specific areas sampled represent sub-regions of contagious patches of suitable habitat within the same mountain range and are practically identical with respect to their terrain, human impact and overall habitat characteristics (see Supplementary Appendix S1, for comparison of landscape characteristics). Second, given snow leopard life history, populations are unlikely to vary dramatically over a two-year period. For example, Sharma et al. (2014) demonstrated through multiple years of camera trapping that populations of snow leopards in Mongolia were constant across each of the four years sampled. Primary wild prey for snow leopards in the study area are Siberian ibex (*Capra sibirica*), blue sheep (*Pseudois nayaur*) and Marcopolo sheep (*Ovis ammon polii*). Annual wildlife census data available with the provincial wildlife department and published data^40–42^, suggest that populations of these mountain ungulates are stable for several years and did not experience any significant annual fluctuations.

## Results

### Trapping success

Snow leopards were detected at ten unique stations in each year, but capture success was higher in the compact design compare to the diffuse design: 34 events, 1.54 captures/100 trap-nights and 21 events, 0.66 captures/100 trap-nights, respectively (Table 1). The compact design, which comprised of 38 traps, produced 562 photos of snow leopards, 27 independent capture events (treating photos within 24 h of a detection as non-independent), and only four unique individuals (Table 1). These four individuals were detected 2, 7, 8, and 17 times at 2, 5, 6, and 7 spatial locations, respectively. The diffused design, which comprised of 44 traps more widely spaced, produced 395 photos of snow leopards, 21 independent capture events, and nine unique individuals (Table 1). Of these nine individuals, one was detected six times, two were detected four times, one seen twice, and the other eight were detected at only one location. One individual that was detected four times was seen at four unique locations whereas all other individuals were detected at a single location. Interestingly, there were no common individuals captured between the two sessions, despite the spatial overlap.

**Table 1:** Summary of snow leopard photo-captures in Hunza-Nagar districts, Gilgit-Baltistan, Pakistan, during two camera trapping sessions.

Design	CE	RE	SR	Traps	Traps with captures	C/100 T nights	C/T
Compact	34	30	10	38	10	1.54	0.8
Diffuse	21	12	3	44	10	0.66	0.48

*C* captures, *T* traps, *CE* capture events, *RE* recapture events, *SR* spatial recapture events.

### Density estimation

**Figure 2:**
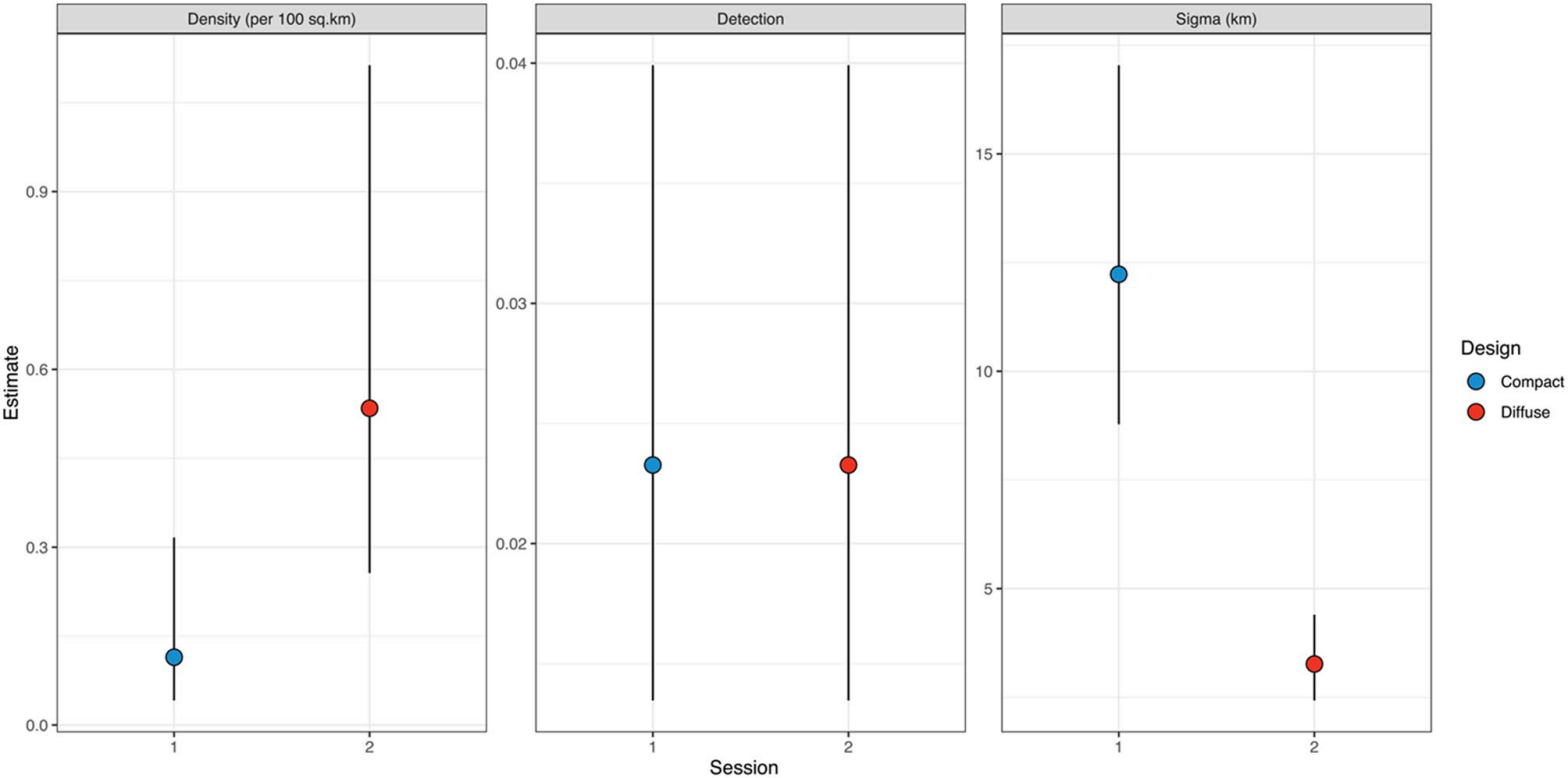
Effect of design on SCR parameter estimates in camera trap study of snow leopards in Hunza-Nagar districts, Gilgit-Baltistan, Pakistan. Bars indicate 95% Confidence Intervals.

The top ranked model suggested that estimates of density and space use ($\sigma$) varied by session, but that encounter rate ($\lambda _{0}$) was constant (Table 2, Fig. 2). It is worth noting again that in this study, we expect the snow leopard population to be the same in both years (2016 and 2018) and, therefore, any differences in parameter estimates are likely to be a result of differences in the sampling design. As such, we find that SCR-based estimates of density and space use are indeed sensitive to the design in this low-density species. The fully session-specific model, i.e., without the constant $\lambda _{0}$ constraint, received similar AIC-support although was ranked second behind the simpler model of one less parameter. This suggests parameter redundancy (Table 2)^43^. To illustrate the differences in estimates of SCR model parameters we report the results from the top ranked model.

**Table 2:** Comparison of spatially explicit capture-recapture (SCR) models tested on camera trap data of snow leopards to evaluate the effect of sampling design.

Model	logL	K	AIC	dAIC	Weight	CumWt
D(~ session) p(~ 1) sig(~ session)	132.37	5.00	274.73	0.00	0.53	0.53
D(~ session) p(~ session) sig(~ session)	131.73	6.00	275.47	0.74	0.37	0.90
D(~ 1) p(~ 1) sig(~ session)	135.53	4.00	279.07	4.34	0.06	0.96
D(~ 1) p(~ session) sig(~ session)	135.06	5.00	280.12	5.39	0.04	1.00
D(~ 1) p(~ session) sig(~ 1)	141.40	4.00	290.81	16.07	0.00	1.00
D(~ session) p(~ session) sig(~ 1)	140.66	5.00	291.31	16.58	0.00	1.00
D(~ 1) p(~ 1) sig(~ 1)	146.77	3.00	299.54	24.81	0.00	1.00
D(~ session) p(~ 1) sig(~ 1)	146.73	4.00	301.46	26.73	0.00	1.00

*D* density, *p* detection probability, *sig* sigma ($\sigma )$, *asu* cost surface.

Support for the session-specific space use model suggests that sampling design influences estimates of $\sigma$. Estimated space use was higher for the compact design: $\hat{\sigma }$ = 12.23 (95% CI 8.78–17.04), than in the diffuse design: $\hat{\sigma }$ = 3.27 (95% CI 2.45–4.35, Fig. 2, Table 3). Converting these estimates to a 95% home range size ($H_{{95}} = \pi ~\left( {\sigma \sqrt {5.99} } \right)^{2}$) gives estimates of 2764.23 km^2^ and 201.22 km^2^ for the compact and diffuse designs respectively. Given the lack of support for a session effect on detectability (Table 2), the baseline encounter rate ($\lambda _{0}$) from the top ranked model was 0.023 (95% CI 0.013–0.040) (Fig. 2, Table 3).

**Table 3:** Parameter estimates of SCR models tested on snow leopard data in Hunza-Nagar districts, Gilgit-Baltistan, Pakistan.

Model	Session	D	N	${{\uplambda }}_{0}$	σ
m5	Compact	0.11 (0.06)	6.95 (3.60)	0.023 (0.006)	12.23 (2.07)
	Diffuse	0.53 (0.20)	32.42 (12.14)		3.27 (0.50)
m7	Compact	0.11 (0.06)	6.82 (3.50)	0.032 (0.014)	11.94 (1.76)
	Diffuse	0.55 (0.21)	33.16 (12.75)	0.017 (0.007)	3.58 (0.66)
m3	Compact	0.25 (0.08)	15.50 (4.66)	0.023 (0.006)	10.99 (2.00)
	Diffuse				3.62 (0.56)
m6	Compact	0.25 (0.08)	15.12 (4.53)	0.03 (0.013)	11.01 (1.85)
	Diffuse			0.017 (0.007)	3.98 (0.77)
m2	Compact	0.23 (0.08)	13.85 (4.84)	0.028 (0.013)	8.57 (1.87)
	Diffuse			0.006 (0.002)	
m4	Compact	0.14 (0.08)	8.74 (4.72)	0.031 (0.015)	8.85 (1.65)
	Diffuse	0.30 (0.12)	18.32 (7.10)	0.005 (0.002)	
m0	Compact	0.26 (0.08)	15.81 (4.89)	0.014 (0.003)	6.68 (0.90)
	Diffuse				
m1	Compact	0.23 (0.12)	14.06 (7.35)	0.014 (0.003)	6.69 (0.90)
	Diffuse	0.28 (0.10)	16.70 (5.98)		

*D* density/100 km^2^, *Ν* population, $\lambda _{0}$ detection probability, *σ* spatial scale parameter.

Standard error is provided in parenthesis.

**Figure 3:**
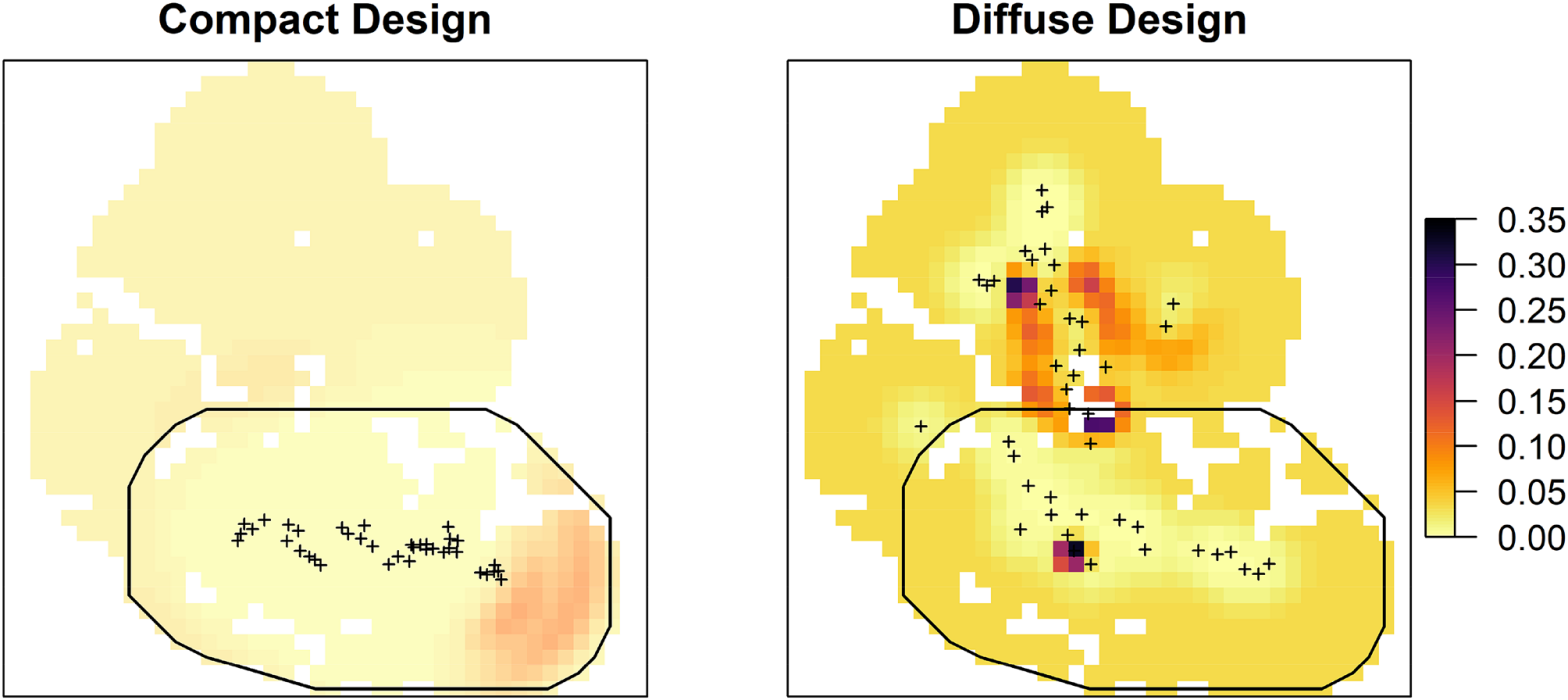
Spatial pattern of snow leopard density (animals/100 km^2^) in Hunza-Nagar districts, Gilgit-Baltistan, Pakistan. Plus sign represents trap locations. Black polygon marks area of compact design.

Estimated density in the compact design was 0.12 animals/100 km^2^ (95% CI 0.041–0.316) with a population estimate for the total area of 6.95 (95% CI 2.5–19.19), and for the diffuse design was 0.534 animals/100 km^2^ (95% CI 0.256–1.113) with a population estimate for the total area of 32.42 leopards (95% CI 15.56–67.55, Table 3, Fig. 3). Remarkably, estimated density was 5 times higher in the diffuse design.

Assuming a stable population over the 2 year sampling period, where sampling was conducted for a similar duration in the same season, SCR parameters estimates were sensitive to differences between the compact and diffuse designs. Estimates of baseline encounter rate were similar across the two design, density was five times higher for the diffuse design, and, conversely, estimated space use was three times higher for the compact design (Table 3, Fig. 2).

## Discussion

Sampling design is a monitoring challenge that is especially pervasive issue for large carnivores because of their low densities and elusive behavior. In this study, we use two years of snow leopard camera trapping data from the same areas in the high mountains of Pakistan, but with vastly different camera configurations, to demonstrate that estimates of density and space use can be sensitive to the configuration of a trapping array. The compact design, one in which cameras are placed much closer together than generally recommended, and therefore have lower spatial coverage, resulted in fewer individuals observed but more recaptures, and estimates of density and space use were inconsistent with expectations for the region. In contrast, the diffuse design, one with larger spacing and spatial coverage, more consistent with general recommendations, detected more individuals, had fewer recaptures, but generated estimates of density and space use that were more in line with expectations. While we acknowledge that both designs produce data that are at the limit of acceptability for spatial capture-recapture, it is worth noting that these data do resemble typical sample sizes for large carnivores^25^, including snow leopards^14^. As such, our aims were twofold: (1) to show that the relative differences between estimates are intuitive given the characteristics of the two designs, and (2) to promote a wider discussion about the importance of study design using an empirical demonstration that has large conservation implications.

Existing recommendations suggest that biased estimates of density can be avoided when two criteria are satisfied^30–32^, although field validation of such assumptions remains somewhat limited (but see^44^). The first recommendation is that the trapping array is at least as large as one home range, although for snow leopards, Suryawanshi et al. (2019) suggest a minimum sampling area of 573 km^2^. The second recommendation is that trap spacing is at most the maximum movement distance (the ‘2-$\sigma$’ rule of thumb), although there is little mention in the literature about the minimum distance.

In the northern parts of the snow leopard range, typical home ranges are 207 km^2^^34^. Here, for illustration purposes, we compare the 95% home range areas derived from the estimated encounter model, openly acknowledging that home range size estimates from SCR generally considered to be proportional to home range size^15^ and can be biased when circular home ranges are assumed^45^. Using the transformation $\pi r_{{95}}^{2}$, where $r_{{95}} = 2.45\sigma$, 95% home range estimates can be generated from both designs. The compact design was about the size of home range (253 km^2^, ratio: 1.2) but smaller than the suggested 573 km^2^, with 1 km trap spacing that is well below the 2-$\sigma$ (8 km) value. The diffuse design was about an order of magnitude larger than a home range (2030 km^2^, ratio: 9.8) with trap spacing closer to, but still lower than, 8 km. While both designs appear to adhere to existing general recommendations, they produce vastly different estimates of density, and the smaller area produced density estimates lower than the larger area, contrary to the behavior predicted by Suryawanshi et al. (2019). This highlights an important knowledge gap in the design recommendations for SCR, such that how strongly spatial variation in density can influence parameter estimates, even in studies that adhere to existing design recommendations. This is especially true in cases where the species exists at low densities and has very low detectability, resulting in the least precision and much higher bias that what has been considered in past simulation studies^30–32^.

In the context of existing ecological knowledge about snow leopards, however, the results of diffuse design are in fact consistent with expectations. For example, the species is known to be highly elusive (yields very low detection, 0.0007–0.01)^5,46^ and live in extremely low densities in rugged terrain across huge landscapes (resulting in very large $\sigma$ (3.8–7.27)^46–48^. Moreover, number of encounters in the current study, are far less than what is considered to be optimal for density estimation (minimum 10–20 individuals^17^, and minimum 10 recaptures for SCR^49^). This data, however, resembles typical sample size in large carnivores^25^. Such as, capture rates in snow leopards are as low as 3/1000 trap-nights^4^, and average unique captures in past camera trap are 7.5 (range 1–20) individuals^24^.

The SCR models predict animal’s movement beyond the trapping array, based on the spatial information of detectors and capture frequencies. Sollmann et al. (2012) acknowledge that movement can be overestimated in a small study area where capture probability at all observed distances is almost equal. This argument partially explains the phenomenon we observed in the compact design because captures across several trap stations in this design were uniform. However, neither the overall study area size (253 km^2^), nor the distances between the spatial captures were small. For instance, maximum observed distances (aerial) moved by four identified individuals were 14–35 km. Equal detections of these individuals across several trap stations could be explained by landscape characteristics—steep slopes, cliffs and narrow valleys and watersheds. Topography constrains animals’ movement to trails along narrow valleys, consequently increasing detections over long distances along the same path. On the other hand, such terrain limits animal movement in adjacent watersheds due to steep peaks in between, thus allowing movement along one axis while restricting it along other axes. SCR models assume circular home ranges and overestimate $\sigma$ enormously due to the following: (i) the distance-based detection function inflates spatial movement along the axis of animal’s spatial recaptures, (ii) predicts the same level of movement along other axes, disregarding the fact that large-scale movement is constrained to a single axis, only. Thus, the upward bias in $\sigma$ a in compact design, has induced negative bias in density estimates^50^. On the other hand, estimate of density (0.53 animals/100 km^2^, 95% CI 0.26–1.13) in diffuse design was in line with expectations. For example, average densities for snow leopards reported in studies that deployed direct methods (camera trapping, genetics) are 0.15–6.5 per 100 km^2^^24^.

Monitoring a wide-ranging, elusive, and rare species is challenging and often leads to large carnivore researchers opting for more compact camera configurations in an attempt to maximize the encounter probabilities of a smaller number of individuals^14,31^. This clearly limits the spatial extent that can be sampled, and here we demonstrate empirically that sampling a small area approximately the size of a single home range is subject to biases resulting from very few individuals on the landscape with very low encounter probabilities. The concern is that the number of individuals in a low density landscape exposed to capture using a smaller and denser array can be far lower than deemed sufficient for estimation^51^. Configuration of traps is considered a critical factor in SCR studies, and appropriate configuration of trapping array can potentially solve issues related to spatial coverage and trapping spacing. Clustered sampling can be a promising approach for survey surveying large mammals over a wider landscapes^52^, as it yields reliable estimates by increasing chances of spatial recaptures and unique captures simultaneously^32,52,53^. While dealing with low density animals, precision can be improved by increasing number of detectors to increase detections, sampling larger areas and ensuring cross-cluster sampling of individuals^52,54^.

The evolution of population estimation for large mammals has been facilitated by the simultaneous development of technology, such as camera traps^55^, and statistical methods (SCR^21,36^). However, the emerging consensus is that study design considerations shall remain an important area of research and development (Efford and Boulanger 2019). This subject has received some attention recently and tools have emerged to generate^13,15^ and evaluate^20^ proposed designs. We advocate strongly that these optimization and simulation tools are adopted in a desk-based pre-survey phase of a camera trap study, especially when monitoring elusive species over large areas in difficult terrain. For example, Dupont et al. (2020) provide an optimization tool that can generate optimal designs in heterogeneous landscapes with incomplete accessibility and with resource constraints (e.g., number of cameras available), and Dupont et al. (2020) and Durbach et al. (2020) demonstrate that a range of objective functions can be used to generate optimal designs based on the objectives of the study. Once a design has been identified, the simulation tool developed by Efford and Boulanger (2019) can be used to understand the properties of that design (e.g., bias and precision). However, these approaches require at least some knowledge of what the SCR parameter values are which highlighting the critical role of using and sharing the vast existing expertise and empirical knowledge that has accumulated over decades of snow leopard monitoring.

## Supplementary Information

Supplementary Information.
