# Supplementary material for: An empirical demonstration of the effect of study design on density estimations
Source: Sci Rep. 2021 Jun 23;11:13104. doi: 10.1038/s41598-021-92361-2 (PMC8222225; doi:10.1038/s41598-021-92361-2)
Supplement: Supplementary file 1 — Supplementary Information. [file 41598_2021_92361_MOESM1_ESM.docx]

**An empirical demonstration of the effect of study design on density estimation.**

Muhammad Ali Nawaz^1*^, Barkat Ullah Khan^2,3^, Amer Mahmood^2^, Muhammad Younas^3^, Jaffar ud Din^3,4^, Chris Sutherland^5,6^

^1^Department of Biological and Environmental Sciences, Qatar University, Doha, Qatar

^2^Department of Zoology, Quaid-i-Azam University, Islamabad, Pakistan

^3^Snow Leopard Foundation, Islamabad, Pakistan

^4^Snow Leopard Trust, USA

^5^Department of Environmental Conservation, University of Massachusetts, Amherst, MA 01002

^6^Centre for Research into Ecological & Environmental Modelling, University of St Andrews, Scotland.

_______________________________________________________________________________

^*^Corresponding author

e-mail addresses: [nawazma@gmail.com](mailto:nawazma@gmail.com) (MA Nawaz) ^*^, [barkat@slf.org.pk](mailto:barkat@slf.org.pk) (BU Khan), [amermahmoodkust@gmail.com](mailto:amermahmoodkust@gmail.com) (A Mahmood), [younas@slf.org.pk](mailto:younas@slf.org.pk) ( M Younas), [jaffar@slf.org.pk](mailto:jaffar@slf.org.pk) (JU Din), [younas@slf.org.pk](mailto:younas@slf.org.pk) (M Younas), [css6@st-andrews.ac.uk](mailto:css6@st-andrews.ac.uk)

(C Sutherland).

**Supporting Information:**

Appendix S1**:** Comparison of landscape characteristics in areas covered by each of the two camera trapping designs. Values are the average with the range in parenthesis and are computed in ArcGIS.

|  | Compact Design | Diffuse Design |
| --- | --- | --- |
| Elevation (m) | 4661.59 (2073 to 7792) | 4461.51 (1949 to 7792) |
| Slope (degrees) | 29.25 (0 to 75.98) | 30.56 (0 to 79.43) |
| Terrain Ruggedness Index | 21.99 (0 to 293.07) | 22.90 (0 to 330.24) |
| Settlements density (per 100 km^2^) | 0.0137 (0 to 0.07) | 0.0129 (0 to 0.07) |
| Road-density (per 100 km^2^) | 0.0061 (0 to 0.083) | 0.0265 (0 to 0.198) |

Appendix S2: Images of Bharpu (A, B) and Baltoro 1 (C, D) snow leopards captured in Session 1 and 2, respectively during camera trapping in Hunza-Nagar districts, Gilgit-Baltistan, Pakistan

| 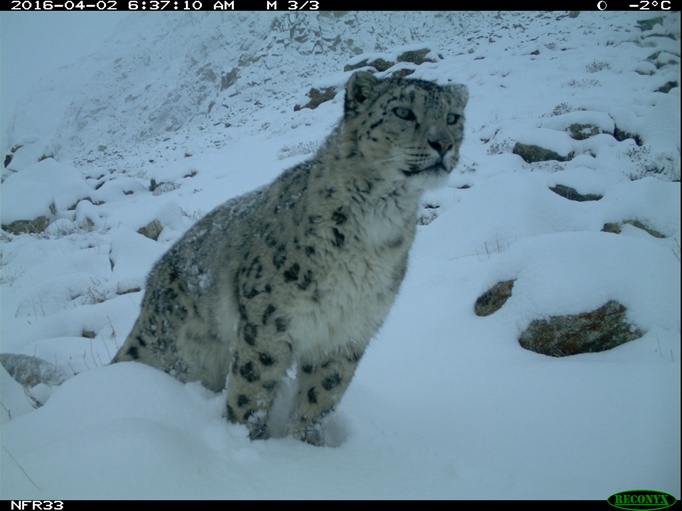 | 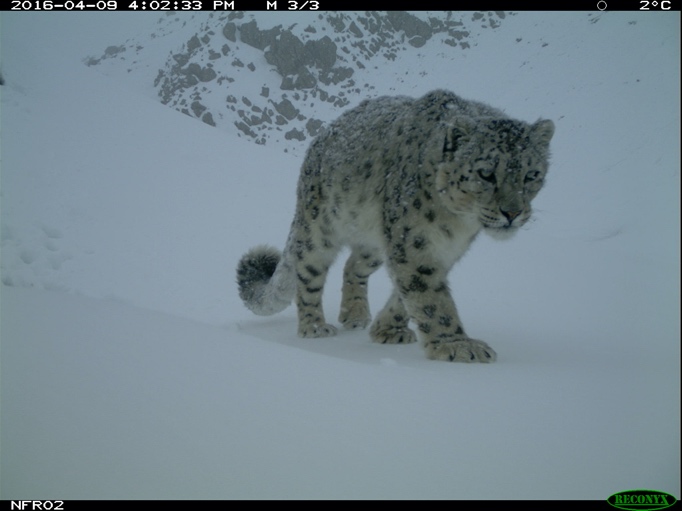 |
| --- | --- |
| (A) | (B) |
| 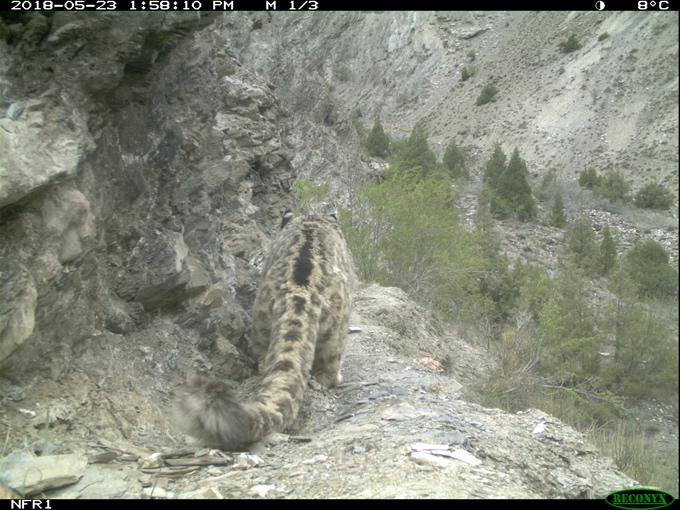 | 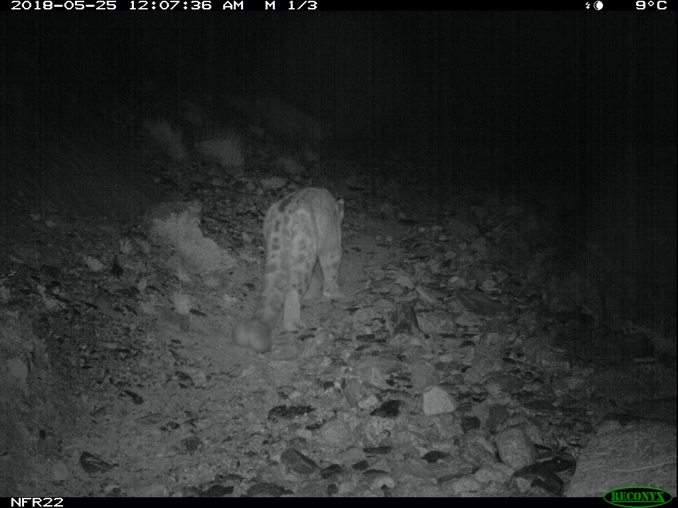 |
| (C) | (D) |
